# Mass spectrometric detection of KRAS protein mutations using molecular imprinting†

**DOI:** 10.1039/d1nr03180e

**Published:** 2021-11-29

**Authors:** Rachel L. Norman, Rajinder Singh, Frederick W. Muskett, Emma L. Parrott, Alessandro Rufini, James I. Langridge, Franscois Runau, Ashley Dennison, Jacqui A. Shaw, Elena Piletska, Francesco Canfarotta, Leong L. Ng, Sergey Piletsky, Donald J. L. Jones

**Affiliations:** Leicester Cancer Research Centre, Leicester Royal Infirmary, University of Leicester Leicester LE1 5WW UK Djlj1@le.ac.uk; Department of Molecular and Cell Biology, University of Leicester LE1 7RH Leicester UK; Leicester Institute of Structural and Chemical Biology, University of Leicester LE1 7RH Leicester UK; Waters Corporation Wilmslow SK9 4AX UK; MIP Diagnostics, The Exchange Building Colworth Park MK44 1LQ Bedford UK; School of Chemistry, University of Leicester, University Road Leicester LE1 7RH UK; Department of Cardiovascular Sciences, University of Leicester and National Institute for Health Research Leicester Biomedical Research Centre, Glenfield Hospital Leicester LE1 7RH UK

## Abstract

Cancer is a disease of cellular evolution where single base changes in the genetic code can have significant impact on the translation of proteins and their activity. Thus, in cancer research there is significant interest in methods that can determine mutations and identify the significant binding sites (epitopes) of antibodies to proteins in order to develop novel therapies. Nano molecularly imprinted polymers (nanoMIPs) provide an alternative to antibodies as reagents capable of specifically capturing target molecules depending on their structure. In this study, we used nanoMIPs to capture KRAS, a critical oncogene, to identify mutations which when present are indicative of oncological progress. Herein, coupling nanoMIPs (capture) and liquid chromatography-mass spectrometry (detection), LC-MS has allowed us to investigate mutational assignment and epitope discovery. Specifically, we have shown epitope discovery by generating nanoMIPs to a recombinant KRAS protein and identifying three regions of the protein which have been previously assigned as epitopes using much more time-consuming protocols. The mutation status of the released tryptic peptide was identified by LC-MS following capture of the conserved region of KRAS using nanoMIPS, which were tryptically digested, thus releasing the sequence of a non-conserved (mutated) region. This approach was tested in cell lines where we showed the effective genotyping of a KRAS cell line and in the plasma of cancer patients, thus demonstrating its ability to diagnose precisely the mutational status of a patient. This work provides a clear line-of-sight for the use of nanoMIPs to its translation from research into diagnostic and clinical utility.

## Abstract 2

We show using Molecular imprinted Polymers (MIPs) and LC-MS/SRM that we can identify the KRAS mutation in cancer patients plasma as well as carry out epitope discovery for drug target evaluation.

## Graphical abstract

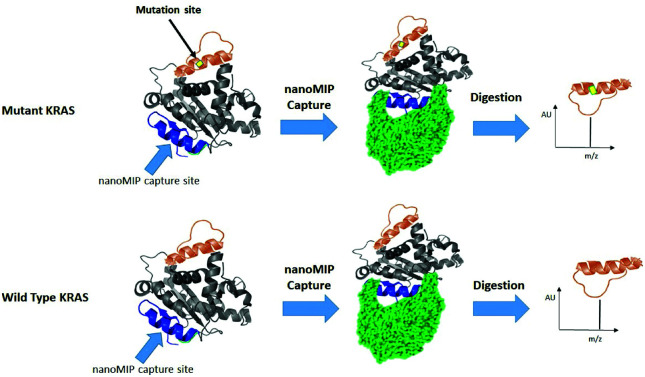

## Introduction

Precision medicine in cancer therapy requires highly specific, reproducible and quantitative methods for the identification and quantitation of molecules that indicate the mutational status of a patient, thus help informing the clinician about the most appropriate therapy.^1–3^ Ideally, these methods should be minimally invasive, reproducible, and cost effective and avoid potentially complicated procedures such as tissue biopsy.^4^ Current methods to determine the mutation status of a patient rely on polymerase chain reaction (PCR) of tissue samples, which can take up to a week to obtain results.^5^ Thus, there is a clear clinical need to establish a method which is non-invasive, highly specific and will provide results in a reasonably short turnaround time.

KRAS (Kirsten RAt Sarcoma) is a member of the RAS family of genes^6^ and represents a classic oncogene that has clinical utility.^7^ The three RAS proto-oncogenes encode four proteins: NRAS, HRAS and the two splice variants KRAS4A and KRAS4B. The four proteins have high sequence homology, mainly differing at C terminal residues 165-188/9, which is also known as the hypervariable region. Ras proteins act as molecular switches by alternating between an active GTP-bound state and an inactive GDP-bound conformation. Ras proteins constitute major players in the MAPK pathway and are activated in response to ligand binding to Epidermal Growth Factor Receptor (EGFR).^8^ Ligand binding to EGFR leads to dimerisation of the receptor, changing its conformation so that growth factor receptor-bound protein 2 (GRB2) can bind. GRB2 recruits son of sevenless (SOS), a guanine nucleotide exchange factor (GEF) which dissociates guanine nucleotides from KRAS, allowing the more abundant GTP to bind and activate KRAS.^9,10^ Active KRAS binds to and enables activation of the BRAF kinase, which, in turn, stimulates cell division *via* the MEK-ERK signalling cascade.

At the clinical level, mutation of KRAS occurs in nearly 30% of all cancers and thus provides a clear target for diagnostics and targeted therapies.^11^ The most common mutation in KRAS is at codon 12, which in the wild type (WT) protein is occupied by a glycine (G) residue. When mutations occur, the glycine can alter to a number of alternatives including cysteine, aspartic acid, valine, alanine and serine. Other mutations occur at position 13, 61 and 146.^12^ Clinically, KRAS mutation status is widely used for colorectal and lung cancer but has utility in pancreatic and thyroid cancers.^13^ A number of methods can be used including pyrosequencing, Sanger sequencing, high-resolution melting analysis, single-strand conformation polymorphism analysis and allele-specific PCR methods that target mutation hotspots in KRAS exons 2 and 3 are currently employed in clinical testing of KRAS mutations.^13^ Attempts have been made to use immune capture prior to MS with reasonable levels of success in tissues but not plasma, preventing their application in a non-invasive test.^14–16^

Epitope discovery seeks to identify the region of a protein that binds most strongly to an antibody. There are many approaches, all of which are quite technically challenging. Modern methods for epitope discovery include Cryo-Electron Microscopy (CryoEM),^17^ Mass Spectrometry (*e.g.* Hydrogen–Deuterium Exchange (HDX)^18^ and MALDI-MS using crosslinking approaches^19^), and Nuclear Magnetic Resonance (NMR)^20^ to determine sites of interaction. Other biological methods include site-directed mutagenesis^21^ or ELISA inhibitor competition assays.^22^ All these methods provide relatively high resolution determination of epitopes but are all technically challenging, and very time consuming. Most importantly, they all require an antibody, the production of which can take months.

Molecularly imprinted polymers (MIPs) are synthetic binders, able to capture specific molecules by means of their target-specific binding sites. They are formed by polymerising a mixture of monomers in the presence of a target molecule which acts as a template.^23^ Once the template has been removed, it leaves a cavity in the polymer which retains its configuration and has the correct shape and functional group orientation to bind the target molecule with specific affinity.^24^ As they have memory of the size shape and functional groups, they can effectively be reused. They have shown to be highly selective in a number of applications for small molecules, peptides and proteins.^25–27^ They have had many different applications, as sensors,^28^ purification tools^29,30^ and in protein production.^31^ More recently, MIPs have been used in diagnostic and sensor applications providing a superior alternative to antibodies thanks to their robustness and reproducible synthesis that does not require the use of animals.^26,32,33^ Moreover, due to their high affinity, they allow for preconcentration of the analyte in question which provides a distinct advantage in scenarios where the analyte is expected to be at very low concentrations within a complex matrix.

In this paper, we describe the synthesis and application of MIPs as a diagnostic tool to recognise the C-terminus of the KRAS protein, which is conserved in both the WT and mutated protein. We use a synthetic mimic that contains the C-terminus of KRAS to generate the nanoMIPs. It is at the N-terminus that the clinically relevant mutations exist. The combination of MIP-assisted capture and mass spectrometry was used to distinguish between WT and mutated forms of KRAS, and we successfully tested this approach in three distinct biological systems demonstrating the utility of this approach for the diagnosis of cancer. We were also able to apply the nanoMIPs as a tool for epitope discovery, by identifying the three main epitopes of KRAS.

## Results

**Fig. 1:**
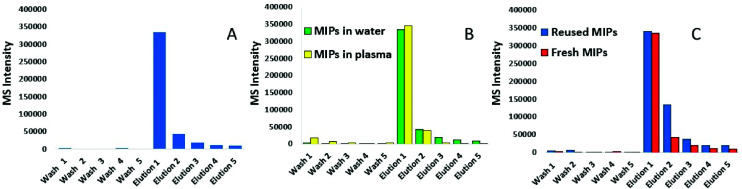
Method development of KRAS-capturing nanoMIPs; LC-MS was used to analyse the: (A) elution of KRAS bridged peptide from nanoMIPs using 10% acetonitrile/10% formic acid (elution is preceded by 5 washes with PBS); (B) elution of KRAS peptide from the nanoMIPs in plasma and water (elution is preceded by 5 washes with PBS), and (C) reusability of MIPs as demonstrated by repeated capture of KRAS.

Following synthesis, nanoMIPs were tested on their ability to capture a synthetic model KRAS molecule (bridged peptide) which contained 35 amino acid sequence including 22 amino acids from the N-terminus of KRAS (shown in red) and 13 amino acid sequence of C-terminus (LVVVGAGGVGKSALTIQLIQNHTPGCVKIKKCIIM). LC-MS was used to analyse the eluant. Fig. 1A shows that the washes are virtually free of peptide, indicating that the PBS step has successfully removed any non-bound peptide. The first elution provided approximately 80% yield of the KRAS peptide, whilst the subsequent elution provides a further 10%. To validate our KRAS-targeted nanoMIPs for applications with clinically relevant material, the same experiment was repeated in human plasma from a healthy volunteer spiked with bridged peptide, mixed with 8 M urea to denature the proteins.

Fig. 1B shows that the recovery of the bridged peptide from plasma by the nanoMIPs was unaffected by the complex matrix which is demonstrated by comparing within the same figure the recovery from water. Moreover, we were able to demonstrate the nanoMIP re-usability by eluting the peptide and then repeating the experiment with another aliquot of plasma. Fig. 1C shows the pattern of intensity between 1^st^ contact, elution followed by a further 2^nd^ contact is very similar.

**Fig. 2:**
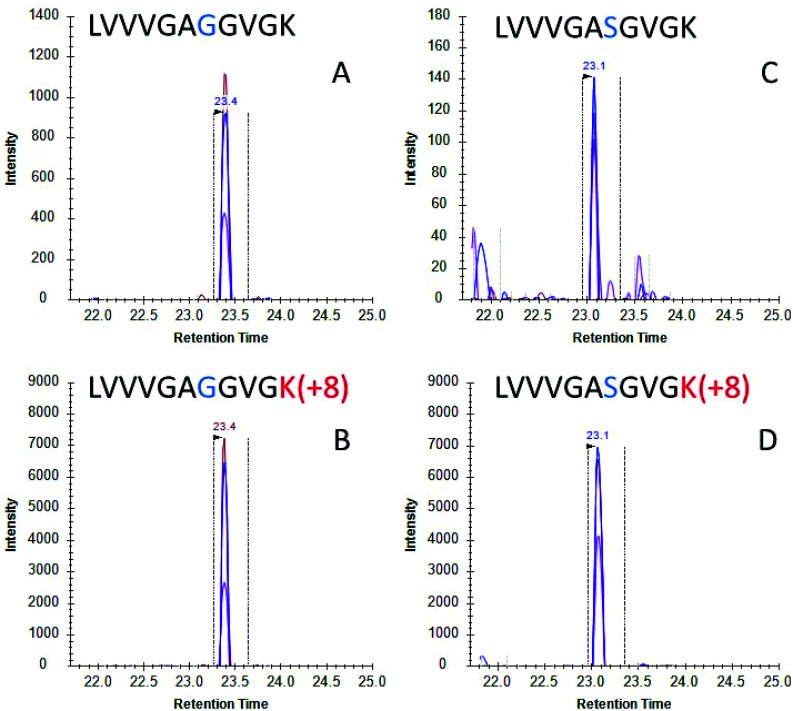
LC-SRM chromatograms for (A) KRAS WT peptide (SRM transitions 478.30 → 743.44, 644.37, 545.30) and (B) stable isotopically labelled standard for KRAS WT peptide (SRM transitions 482.31 → 751.46, 652.39, 553.32) from H1650 cell lysate using on bead digests; and (C) mutant peptide (SRM transitions 493.31 → 773.45, 674.38, 575.31) and (D) stable isotopically labelled standard for G12S (SRM transitions 497.31 → 781.47, 682.397, 583.33) from A549 cell lysate on-bead digests. Multiple transitions sharing the same chromatographic profile are shown for each peptide. Vertical dotted lines indicate an automatically selected peak by the software on predicted parameters.

Once the initial evaluation took place, the nanoMIPs were tested for their efficacy to phenotype cancer cells by detecting KRAS protein in cell culture. Two lung adenocarcinoma cancer cell lines were selected, namely A549 cells which are homozygous for the G12S mutation and H1650 cells which contains WT KRAS. Cells were lysed and the lysate was then incubated with MIP-coated glass beads for 2 hours and the captured proteins tryptically digested. Resultant tryptic peptides were measured using LC-MS/MS (SRM). SRM chromatograms targeting WT KRAS signal in H1650 cells showed several confirmatory transitions for the analyte (Fig. 2A) which eluted with the same retention time as the stable isotope labelled (SIL) standard (Fig. 2B). Conversely in the A549 cells, a homozygous cell line containing the G12S mutation presented several confirmatory transitions for the G12S mutated KRAS protein (Fig. 2C) which co-eluted with the isotopically labelled standard for G12S (Fig. 2D). These data indicate that nanoMIPs are able to select and identify mutations from *in vitro* biological models which would enable investigation of these models and, with appropriate calibration curves, allow the identification and quantitation of mutant KRAS in clinical specimens.

**Fig. 3:**
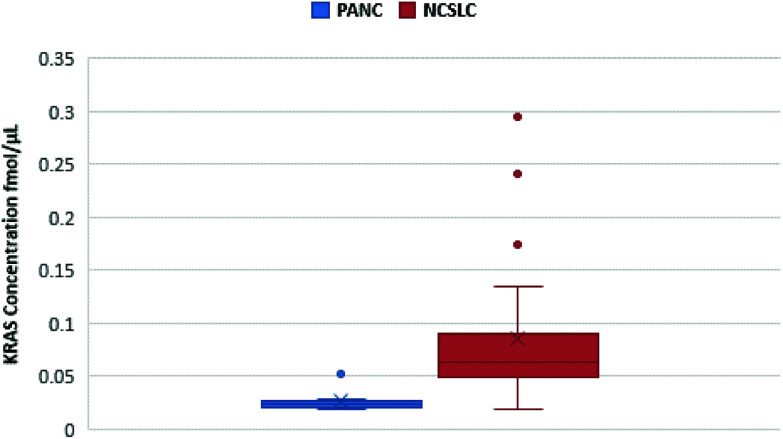
Quantification of KRAS WT 6–16 peptide in pancreatic cancer (blue) and NSCLC (red) plasma samples. The figure shows a spread of KRAS measurements for each cancer.

Notwithstanding our successful detection of KRAS bridged peptides in spiked plasma samples, we reasoned that measurement of endogenous KRAS in such milieu is likely to be hampered due to the inherent low copy number of circulating KRAS proteins.^14^ Thus, we explored whether we could use nanoMIPs to effectively capture and enrich circulating KRAS. Plasma samples from eight treatment naïve patients with pancreatic cancer were obtained from Prof Ashley Dennison and Franscois Runau (University of Leicester). Plasma samples from 25 patients with non-small cell lung cancer (NSCLC) were obtained from Prof Jacqui Shaw (University of Leicester). The genotype of these samples was unknown, but about 90% of pancreatic cancer patients and about a 20% of NSCLC patients harbor KRAS mutations.^11^ Calibration lines were established for 6 of the main mutations and other RAS forms.^34^ For every sample, we were able to detect and measure WT KRAS (Fig. 3), and to the best of our knowledge, this is the first occasion that the gene product of KRAS has been measured in cancer patient plasma using LC-SRM.

**Fig. 4:**
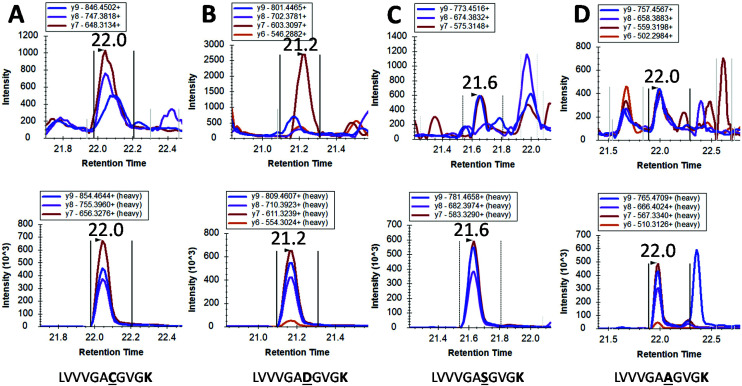
LC-SRM detection of mutated KRAS proteins in pancreatic cancer patients. Each Panel A to D are derived from different cancer patients where a mutation is tentatively identified. Within each panel, the transitions for the analyte are shown on the upper chromatogram whilst the relevant transitions for stable isotope labelled (SIL) standards are shown in the lower chromatogram. Panel A to D show tentative assignments for **G → C**, **G → D**, **G → S** and **G → A** respectively.

However, mutated KRAS forms would inevitably be present in extremely low amounts possibly orders of magnitude less than the WT form. By using our integrated approach, we have detected in 4 pancreatic cancer patients a mutated form (G12C, G12S, G12A and G12D) but at the very limit of detection where a certain degree of doubt would exist in mutation status assignment (Fig. 4). This finding together with the very low concentrations of WT KRAS indicate that the detection of mutated KRAS would require sensitivity beyond the currently achievable limit of detection.

**Fig. 5:**
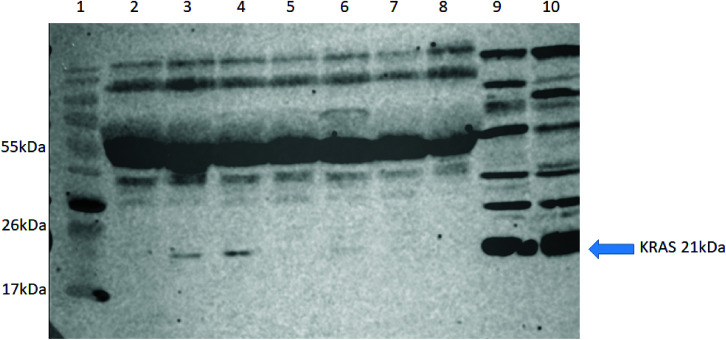
Western blot analysis for detection of KRAS using a KRAS primary antibody. (Lane 1) molecular weight marker, (lanes 2–5,7) NSCLC patient plasma, (lane 6) Pancreatic cancer plasma, (lane 8) a healthy control patient plasma, (lane 9) HCT-N cells and (lane 10) HT-29 cells.

To further validate our approach, we carried out western blot for KRAS using an anti-panKRAS antibody (this antibody captures KRAS without discerning the isoform of the KRAS protein) in seven selected patient samples (5 NSCLC (lanes 2–5, 7), one pancreatic cancer (lane 6) and one healthy control (lane 8) where we had previously identified the WT KRAS by LC-SRM. We also examined two lysates from colorectal cancer cell lines (HCT-N or HT-29) that act as positive controls (lanes 9&10). KRAS was highly expressed in the two positive controls (see G12C) whilst the KRAS protein could only be detected in three out of seven plasma samples, thus confirming our previous finding that MIPs with LC-SRM detect KRAS proteins in plasma samples with better sensitivity than western blot (Fig. 5).

**Fig. 6:**
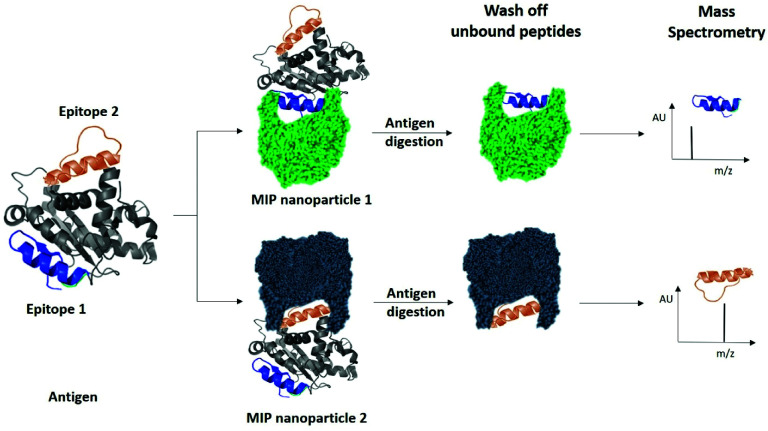
Identification of peptide sequences exposed on protein surfaces using molecular imprinting and mass spectrometry.

Finally, we examined the ability of nanoMIPs to discover epitopes for the whole KRAS protein. The workflow is shown in Fig. 6. We produced recombinant KRAS protein using a KRAS cDNA sequence supplied as a plasmid expressed in a DH10B TonA *E. coli* host (all details described in ESI†). The purified recombinant KRAS protein was used as a template to generate nanoMIPs using a modified solution imprinting process.^23^ The protein was then tryptically digested whilst still bound to the nanoMIP. Sections of the protein that were not directly protected by the MIP binding site and underwent digestion were washed away. Only the bound peptides of KRAS that were within the binding site of the nanoMIPs would represent epitopes. The epitope peptides were eluted from the nanoMIPs using hot water (95 °C) and identified using LC-MS/MS. In total, three peptides were identified by this epitope discovery process (see Table 1). The experiment was repeated independently three times and the same three peptides emerged. When the recombinant protein was tryptically digested in the absence of MIPs, a set of tryptic peptides resulted, many of which were stronger in intensity than the three putative epitope peptides. This result indicates that the peptides identified as epitopes are not merely the most abundant tryptic peptides, but they reflect the direct and specific interaction between nanoMIPs and KRAS.

**Table 1:** The three peptides identified by the MIP epitope discovery process as potential KRAS epitopes

Epitope	Peptide MW	Sequence	Residues	*R*_*t*_ (min)	Ave. intens.	Peptides listed in the immune epitope database^35–37^
1	955.5935	(K)LVVVGAGGVGK(S)	6–16	29.4	22 603	LVVVGAGGVGKSA/LVVVGAGGVGKSALT
2	1312.678	(R)SYGIPFIETSAK(T)	136–147	36.5	41 018	IPFIETSAKTRQGVDD
3	1383.69	(R)QGVDDAFYTLVR(E)	150–161	37.1	5680	IPFIETSAKTRQGVDD

**Fig. 7:**
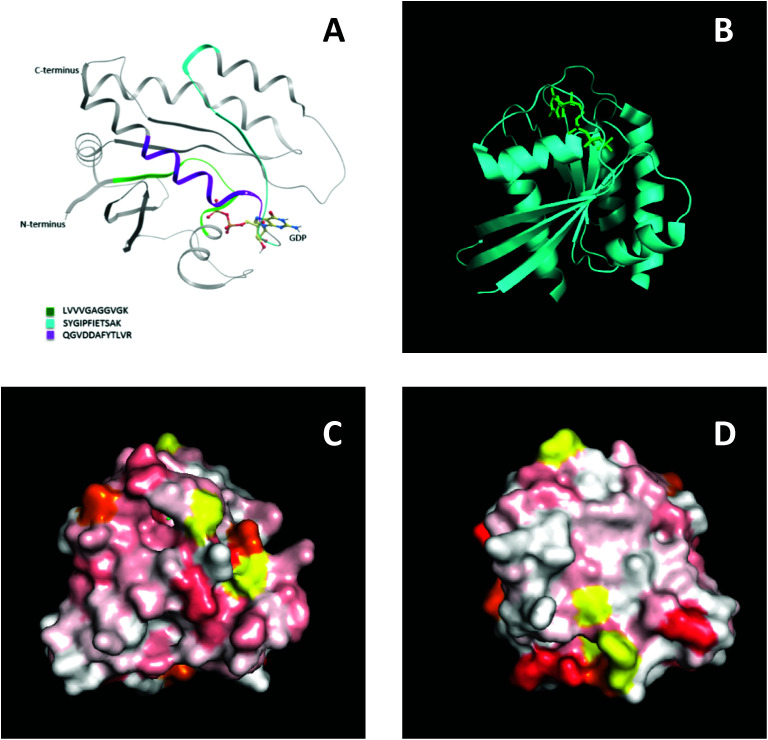
Epitope discovery of nanoMIPs with recombinant KRAS protein (A) epitope sites as identified by nanoMIP directed MS proteomics, (B) crystal structure of KRAS4B (C) surface representation of chemical shift changes on KRAS4B in the same orientation as B. (D) 180° rotation of image C.

To further support this result, NMR spectroscopy was used to map the binding site, or sites, of the MIPs on KRAS. 2D ^15^N-TROSY spectra of ^15^N labelled KRAS were recorded in the absence and presence of MIPs. The effects on the spectra are reported as a chemical shift perturbation analysis (reviewed by Williamson 2013 ^38^) and three candidate epitopes were mapped *in-silico* onto the crystal structure of KRAS4B. The nanoMIPs produced small but significant chemical shift changes in the KRAS spectrum. These were mapped onto the crystal structure as shown in Fig. 7. Orange represents histidine residues and yellow regions are areas where there is no data. The chemical shifts are represented by the white and red areas where white indicates no change and darker red indicates larger chemical shift changes. The NMR data showed that there were multiple binding sites rather than a single site, corroborating the mass spectrometry analysis. The red region, indicating a greater chemical shift at the bottom right-hand corner of Fig. 7D, could match epitope three thus corroborating the MS data and suggesting that the peptides identified by mass spectrometry represent binding sites on the protein.

The three potential epitopes identified using nanoMIPs and mass spectrometry also overlap with confirmed antibody epitopes. Most commercially available antibodies are raised to the N-terminal region of KRAS, which includes the 6–16 peptide which was identified as a binding site by the nanoMIPs and mass spectrometry (Epitope 1). In addition, Santa Cruz Biotechnology, Inc. have developed an antibody raised against a region spanning amino acids 54–189, which includes epitopes two and three. In addition, the three epitopes identified using nanoMIPs and mass spectrometry were all either completely or partially covered by peptides listed in the Immune Epitope Database, as shown in Table 1. Overall, these data collectively show the ability of nanoMIPs and MS to identify protein epitopes.

## Discussion

The identification of a patient's specific mutation could have great opportunity in the clinical management of oncological disease. In particular, informing the application of targeted therapies that depend on specific driver mutations and facilitating early detection of the disease. We have shown in this paper evidence for the application of nanoMIPs generated to a conserved region of the KRAS protein that allows identification of oncogenic mutations. There is a clear clinical need to identify cancer mutations, as an increasing knowledge of the genetic journey of cancer unfolds and consequently the understanding of its impact on patient outcome.^39^ Additionally, assays that are sensitive and amenable to high throughput operation would be desirable. Mass spectrometry is contributing to the field of mutation analysis with a few publications.^40,41^ However, these papers involve significant sample processing which limits the throughputs. The focus of these papers is towards realising information about the number of mutations in relation to RNA-seq data. Previous work has looked at mutant KRAS proteins using Immuno-MS.^14,16^ However, the proposed method here would be implementable in a much shorter timescale, as a consequence of not requiring time consuming antibody production, which allows our approach its potential use as a tool for precision and personalised medicine. The nanoMIP-based capture of KRAS using simple sample preparation lends itself to a high throughput technique such as LC-MS to enable identification of mutant proteins. Although the LC-MS method used within this work utilises nanoflow rates to optimise assay sensitivity at the expense of throughput, recent reevaluation of microflow rates for liquid chromatography have shown that high throughput assays can be achieved without loss of sensitivity.^42,43^ Furthermore, we did demonstrate superior levels of sensitivity compared to antibody-based western blot and were able to detect the WT and tentative mutated forms in plasma of cancer patients. The advantages of nanoMIPs over other capture technologies (selectivity, robustness, rapid turnaround times, batch to batch consistency, no need for cold chain supply and use of animals) provide an ideal sample preparation tool for isolating specific molecules of interest in clinical samples.

The synthesis of the nanoMIPs to the whole protein was carried out in order to discover KRAS epitopes. The experiment was repeated three times and demonstrated reproducible identification of epitopes which were substantiated by NMR. The three epitopes were consistent with those found in the Epitope databases^37^ as shown in Table 1. A study by Bergseng *et al.*^35^ into coeliac disease-associated human leukocyte antigen (HLA) molecules identified peptides eluted from HLA molecules. Epitope 1 was listed as being identified by MaxQuant in peptides eluted from HLA molecules DQ2.5, DQ2.2 and DQ7.5. Epitopes 2 and 3 were partially identified in the immunopeptidome eluted from major histocompatibility complex (MHC) class II molecules in A20 mouse lymphoma cells.^36^ This suggests that the peptides identified using MIPs and mass spectrometry are all part of known immune epitopes.

This work demonstrates that the data obtained using this novel method – recently patented – is analogous to current methods to some extent, and could therefore be a feasible alternative. The method works on the assumption that the polymer protects the region of the protein that it binds to from proteolytic digestion. However, tryptic peptides may be longer than the region enveloped by the polymer. To develop this method further in future, combinations of different proteolytic enzymes could be investigated to obtain complete epitope sequences. This approach provides the possibility of identifying regions of the protein surface which have not been demonstrated to be antigenic *in vivo*, but which may offer improved affinity.

In conclusion we show that the use of nanoMIPs epitope discovery coupled with MS analysis allows epitope identification which, in contrast to the use of antibodies with NMR, is significantly quicker (mainly through the avoidance of antibody production which can take months), does not require *in vivo* experiments and could potentially be automated into a high-throughput screening approach. We also demonstrate mutation detection in clinical samples, which has great potential in the deployment of precision medicine.

## Methods and materials

### NanoMIPs synthesis and characterization

NanoMIPs were synthesised by a solid-phase method which relies on the covalent immobilisation of the primary template (C-terminus of the KRAS) on a solid support (*e.g.* glass beads). *N*-Isopropylacrylamide (NIPAm), *N-tert*-butylacrylamide (TBAm) and *N*-(3-aminopropyl) methacrylamide hydrochloride (amino monomer) were selected as the monomers with *N*,*N*′-methylenebisacrylamide (BIS) used as the crosslinker (described in full in ESI†). These reagents were previously optimised for peptide imprinting.^44–46^ The immobilised template is placed in contact with the monomer solution and polymerisation is chemically initiated and a cross linker introduced to promote the formation of nanoparticles. The nanoMIPs generated are surface imprinted to recognise the C-terminus region of the KRAS protein. The *N*-(3-aminopropyl) methacrylamide hydrochloride (amino monomer) was added to aid immobilisation of MIP nanoparticles onto glass beads.

Following polymerisation, dynamic light scattering (DLS – Zetasizer, Malvern Instruments, UK) was used to assess the average hydrodynamic diameter. The nanoMIPs imprinted against the peptide epitope showed a hydrodynamic diameter of 215.9 nm (±5 (*n* = 6)), whilst nanoMIPs imprinted against a recombinant KRAS protein showed an average particle size of 185.6 nm (±5 (*n* = 6)).

### NanoMIPS testing

NanoMIPs were tested on their ability to capture a synthetic model KRAS molecule (bridged peptide) which contained 35 amino acid sequence including 22 amino acids from the N-terminus of KRAS (shown in red) and 13 amino acid sequence of C-terminus (LVVVGAGGVGKSALTIQLIQNHTPGCVKIKKCIIM). To this end, the nanoMIPs were immobilised onto glass beads (as described in ESI†) and stored at +4 °C. Ten μL of bridged peptide (10 fmol μl^−1^) were added to 350 mg bead immobilised MIPs in a Spin-X column and incubated for 2 hours. The MIPs were washed in PBS to remove non-specifically bound peptides and then washed 3 times with ammonium bicarbonate. Bound peptide was eluted using 200 μL 10% acetonitrile/10% formic acid at RT and analysed using LC-SRM.

### Epitope discovery

The KRAS recombinant protein was generated in a plasmid vector grown in *E. coli* and Ni-HTA his tag purified (full details in ESI†). NanoMIPs were produced using the purified recombinant protein. A solid-phase approach described by Canfarotta *et al.*^23^ was adapted for MIP nanoparticle synthesis. 0.2 mg trypsin (trypsin from bovine pancreas, Sigma) in 5 mL PBS was added to the sample and incubated at room temperature for 96 hours. Unbound KRAS peptides and trypsin were removed by centrifuging for 5 minutes at 4000 rpm then washing twice with 10 mL PBS. The peptides bound to the MIPs were eluted using 1 mL hot water (heated to 95 °C). These potential epitopes were collected by centrifugation on 20 kDa MWCO filters. The hot wash was repeated and both elutions were combined, lyophilised and reconstituted in 20 μL 0.1% formic acid/3% acetonitrile and 20 μL 100 fmol μL^−1^ alcohol dehydrogenase (ADH). The samples were analysed in triplicate on a Waters Synapt G2 mass spectrometer coupled to a Waters Acquity nano-UPLC as described below.

### Cell culture and cell lysis

A549 and H1650 cells were grown in RPMI 1640+ l-glutamine media supplemented with 10% foetal calf serum (FCS) and were passaged when they reached approximately 70% confluency. Cells were not passaged more than 20 times before being replaced by fresh stocks from liquid nitrogen stores. Cells were lysed by addition of 500 μL lysis buffer (8 M urea in 100 mM Tris) then vortexed and sonicated for 15 minutes. Western blot analysis of KRAS proteins are described in ESI.†

### Plasma samples

Plasma samples from 8 treatment naïve patients with pancreatic cancer were obtained from Prof Ashley Dennison and Franscois Runau (University of Leicester. The samples were approved for use by the regional ethics committee (REC number 12/EE/0425)), MHRA and the institutional review board under clinical trial registration number (NCT01019382). Plasma samples from 25 patients with NSCLC were obtained from Prof Jacqui Shaw (University of Leicester. The samples were covered by the ethics approval for the biobank from the regional ethics committee (REC number: 18/EM/0209)). Informed consents were obtained from human participants of both studies. The KRAS status of the pancreatic cancer plasma samples was unknown but, as approximately 90% of pancreatic cancers contain KRAS mutations,^47^ it is assumed that the majority of samples would contain a mutation. Some of the NSCLC patients had their KRAS status determined by genetic testing.

50 μL of each plasma sample was denatured with 350 μL 8 M urea, adjusted to pH 7.8. Denatured plasma was added to 350 mg KRAS4A peptide MIPs, immobilised on glass beads, on a 0.45 μm Spin X centrifugal filter and incubated for 2 hours on a sample rotator. Filters were centrifuged at 8000 rpm for 10 minutes at 4 °C. The filtrate was discarded then the beads were washed with 200 μL PBS three times, followed by 200 μL ammonium bicarbonate, pH 7.8, three times, centrifuging for 5 minutes at 8000 rpm and discarding the filtrate each time. Beads were then washed with 200 μL 10% acetonitrile/10% formic acid twice and the filtrate was collected and stored at −80 °C. Filters were transferred to clean tubes then 200 μL ammonium bicarbonate with 15 mM DTT were added and samples were incubated for 30 minutes at 60 °C. 20 mM IAA was added and beads were incubated for 30 minutes in the dark at room temperature. 16 μg trypsin was added and samples were incubated at 37 °C overnight.

Samples were centrifuged for 10 minutes at 8000 rpm and 20 °C. 1% formic acid was added to the filtrate then samples were incubated for 20 minutes at 37 °C before centrifuging for 10 minutes at 14 000*g*. The supernatant was desalted *via* solid phase extraction which employed empore SPE cartridges. After lyophilisation, samples were reconstituted in 0.1% formic acid/3% acetonitrile with 25 fmol μL^−1^ stable isotope-labelled standards in a matrix of 1 mg mL^−1^ BSA. BSA was added to prevent non-specific binding to the sample vials because the calibration line in solvent displayed reduced peak intensity for many of the peptides. Non-specific binding was eradicated by the addition of digested human plasma as a sample matrix in the standards for the calibration line. In the MIP on-bead digestion samples, most of the patient plasma was removed during the washing steps. A human plasma background would interfere with the analysis as it would contain endogenous RAS protein thus BSA was used as an alternative sample matrix to coat the vials, reducing non-specific binding of the analytes.

### Liquid chromatography mass spectrometry

#### Selected reaction monitoring (LC/SRM)

##### Nano ultra performance liquid chromatography

For LC/SRM analysis, samples were injected onto a Waters NanoAcquity UPLC with a Waters 2G-V/M Symmetry C18 trap column (180 μm × 20 mm, 5 μm) for desalting and chromatographic focussing before elution onto a Waters Acquity HSS T3 analytical UPLC column (75 μm × 250 mm, 1.8 μm) (Waters, Milford, MA). The analytical column temperature was set at 40 °C and the auto sampler temperature was maintained at 6 °C. Trapping occurred for 3 min with 99.9% solvent A and 0.1% solvent B at a flow rate of 5 μL min^−1^. A 30 minutes liquid chromatography gradient was initiated on elution from the trap column. The following gradient was used: 0 min – 3% B, 10 min – 50% B, 10.33 min – 85% B, 21.60 min – 85% B and 22 min – 3% B. The flow rate was set at 0.3 μL min^−1^. Solvent A was LC/MS grade water containing 0.1% formic acid. Solvent B was acetonitrile containing 0.1% formic acid.

##### Mass spectrometry

The Waters Xevo TQ-XS mass spectrometer (Waters, Milford, MA) was operated in positive electrospray ionisation (+ESI) mode. The capillary voltage was set at 2.40 kV and the cone voltage was set at 30 eV. Argon was used as the collision gas and the collision energy was optimised for each peptide by repeated injections using different collision energy voltages. Initially the precursor ion for each peptide was determined by performing a MS scan followed by a product ion scan to identify the most abundant product ions to select as the SRM transitions. Exact *m*/*z* values were extracted from the raw data using Skyline software (MacCoss laboratory).^48^

#### High resolution data independent acquisition (LC-MS/MS (DIA))

##### Nano ultra performance liquid chromatography (NanoUPLC)

Sample analysis was performed using a Waters NanoAcquity UPLC system. The peptides were initially loaded onto a Waters 2G-V/M Symmetry C18 trap column (180 μm × 20 mm, 5 μm) to desalt and chromatographically focus the peptides prior to elution onto a Waters Acquity HSS T3 analytical UPLC column (75 μm × 250 mm, 1.8 μm). Single pump trapping was used with 99.9% solvent A and 0.1% solvent B at flow rate of 5 μL min^−1^ for 3 min. Solvent A was LC-MS grade water containing 0.1% formic acid and solvent B was acetonitrile containing 0.1% formic acid. For the analytical column the flow rate was set at 0.3 μL min^−1^ and the temperature maintained at 40 °C. The 50 min run time gradient elution was initiated as the peptides were eluted from the trap column. The following gradient was used: 0 min – 3% B, 30 min – 40% B, 32 min – 85% B, 40 min – 85% B, 41 min – 3% B and 50 min – 3% B.

##### Nano electrospray ionisation mass spectrometry

The NanoAcquity UPLC was coupled to a Waters Synapt G2 HDMS mass spectrometer. The instrument was operated in positive electrospray ionisation (ESI) mode. The capillary voltage was set at 2.4 kV and cone voltage at 30 V. PicoTip emitters (New Objective, US, 10 μm internal diameter) were used for the nanostage probe. A helium gas flow of 180 mL min^−1^ and ion mobility separator nitrogen gas flow of 90 mL min^−1^ with a pressure of 2.5 mbar were used. The IMS wave velocity was set at 650 m s^−1^ and the IMS wave height at 40 V. During the HDMS^E^ acquisition a low collision induced dissociation (CID) energy of 2 V was applied across the transfer ion guide. For the high CID energy aquisition a ramp of 27 to 50 V was applied. Argon was used as the CID gas. Lockspray provided mass accuracy throughout the chromatographic run using [Glu1]-Fibrinopeptide (GFP) with 785.8427 *m*/*z*. The data was acquired using MassLynx 4.1 and processed to obtain peptide sequences using ProteinLynx Global Server (PLGS 3.0) using a Human Uniprot database (downloaded Nov 2015).

## Author contributions

The manuscript was written through contributions of all authors. RLN, DJ, LLN and SP all designed concept and experiments. RLN ran all experiments. RS and RLN analysed on LC-MS and interpreted results. FWM helped with running the NMR experiments and helped RLN to interpret results. ELP helped run the WB experiments. ELP and AR ran the genotyping of the cancer cell lines. JL helped with analysis of patient samples on LC-MS. FR and AD helped with provision of patient samples and design of experiment. JS helped with provision of patient samples and design of experiment. FC and EP advised on MIPs synthesis, characterization and optimization. All authors have given approval to the final version of the manuscript.

## Conflicts of interest

There are no conflicts to declare.

## Supplementary Material

NR-013-D1NR03180E-s001
