# Supplementary material for: Mass spectrometric detection of KRAS protein mutations using molecular imprinting
Source: Nanoscale. 2021 Nov 29;13(48):20401–11. doi: 10.1039/d1nr03180e (PMC8675027; doi:10.1039/d1nr03180e)
Supplement: NR-013-D1NR03180E-s001 [file NR-013-D1NR03180E-s001.pdf]

Electronic Supplementary Material (ESI) for Nanoscale.
This journal is © The Royal Society of Chemistry 2021

Supporting Information:

Mass spectrometric detection of KRAS protein mutations using molecular imprinting.

Rachel L Norman[1], Rajinder Singh[1], Frederick W. Muskett[2,3], Emma L Parrott[1], Alessandro Rufini[1], James I Langridge[4], Franscois Runau[1], Ashley Dennison[1], Jacqui A Shaw[1], Elena Piletska[5,7],Francesco Canfarotta[5], Leong L Ng[6], Sergey Piletsky[5,7,] Donald JL Jones[1,6]*

[1]Leicester Cancer Research Centre, Leicester Royal Infirmary, University of Leicester, Leicester, LE1 5WW, United Kingdom

[2]Department of Molecular and Cell Biology, University of Leicester, LE1 7RH Leicester, United Kingdom.

[3]Leicester Institute of Structural and Chemical Biology, University of Leicester, LE1 7RH Leicester, United Kingdom.

[4]Waters Corporation, Wilmslow, SK9 4AX, United Kingdom.

[5]MIP Diagnostics, The Exchange Building, Colworth Park, MK44 1LQ, Bedford, UK

[6]Department of Cardiovascular Sciences, University of Leicester and National Institute for Health Research Leicester Biomedical Research Centre, Glenfield Hospital, Leicester, LE1 7RH, United Kingdom

[7]School of Chemistry, University of Leicester, University Road, Leicester, LE1 7RH, UK

Experimental Section:

Materials:

All chemicals and reagents were from Fisher Scientific, Thermo Fisher Scientific and Sigma-Aldrich (UK) unless otherwise stated. Glass beads were obtained from Microbeads AG (Switzerland). The amino monomer, N-(3-aminopropyl)methacrylamide hydrochloride was from Sigma Aldrich (UK). N-(3(trimethoxysilyl)propyl) ethylenediamine was purchased from Acros Organics (Thermo Fisher, UK). KRAS cDNA, an I.M.A.G.E Fully Sequenced cDNA clone (IMAGE ID: 3878884), was obtained from Source Bioscience (Nottingham, UK). Isopropyl β-D-1-thiogalactopyranoside (IPTG) was obtained from Life Technologies Ltd. (Invitrogen, US). BL21-CodonPlus (DE3)-RIL and pLysS competent cells were kindly provided by Dr Aude Echalier-Glazer (University of Leicester). 10 kDa NMWL centrifugal filters were obtained from Millipore (Watford, UK). Spin X pore size 0.45 μm centrifuge tube filters were obtained from Sigma-Aldrich (Gillingham, UK). QIAprep Spin Mini Kit (QIAGEN, Crawley, UK) and DNase/RNase-free distilled water obtained from Fisher Scientific. Endoproteinase GluC was obtained from SERVA Electrophoresis GmbH (Heidelberg, Germany). Stable isotope-labelled peptides were purchased from Pepscan (Lelystad, The Netherlands) whilst unlabelled peptides were custom synthesised by Genecust (Ellange, Luxembourg) unless otherwise stated. Water was Optima LC-MS grade (Fisher Scientific). A549 and H1650 cells were obtained locally (RKCSB, UoL). A549 contains a KRAS G12S mutation and H1650 contains KRAS WT.

Recombinant KRAS generation: The KRAS cDNA was supplied as a plasmid expressed in a DH10B TonA E.coli host, thus, the E.coli needed to be cultured in a liquid media, lysed and the DNA extracted. A sterile pipette tip was used to scrape the surface of the agar in the cDNA vial.

This was then spread onto an LB agar plate containing 100 μg/mL ampicillin as the plasmid conferred ampicillin resistance. The plate was incubated overnight at 37 °C then stored at 4 °C. A single colony was scraped off the agar plate then ejected into a falcon tube containing 10 mL Miller LB broth with 100 μg/mL ampicillin. The tube was incubated in a shaking incubator at 37 °C overnight. After 500 μL culture was removed to make glycerol stocks, the remaining culture was split into Eppendorf tubes and centrifuged at 9000 rpm for 3 minutes. The supernatant was discarded then the pellets were reconstituted in 50 μL buffer P1 from the QIAprep Spin Miniprep Kit and combined to give a total of 250 μL in each tube. cDNA was extracted from the cells by following the provided QIAprep protocol, omitting the LyseBlue reagent. The DNA was eluted from QIAprep spin column with 50 μL buffer EB. DNA concentration was measured using a Nanodrop 1000 spectrophotometer. 200 ng/μL cDNA was submitted to PROTEX (University of Leicester) along with forward and reverse primers (Sigma) for cloning into a pLEIC-01 vector. The plasmid was sequenced at the Protein Nucleic Acid Chemistry Laboratory (PNACL, University of Leicester) to check that the cloning was successful and there were no spontaneous mutations.

Transformation: Two different cell lines were used for small scale test expressions to see which one would successfully express KRAS and have the highest yield. Two different strains of E.coli were tested with and without detergent. Competent BL21-CodonPlus (DE3)-RIL and pLysS cells were thawed on ice. 1 μL KRAS1-169 plasmid was added to 19 μL cells and left on ice for 15 minutes. Cells were subjected to heat shock at 42 °C for 45 seconds then returned to ice for 5 minutes. 100 μL LB media (with no antibiotic) was added to each tube and incubated at 37 °C in a shaking incubator for 1 hour. 70 μL cells were pipetted onto LB agar plates containing ampicillin and spread with a sterile spreader. The plates were incubated at 37 °C overnight.

For the starter cultures, four Falcon tubes were filled with 5 mL LB containing ampicillin. Colonies were picked from the plates using a pipette tip and dropped into two tubes for each cell line which were then incubated overnight at 37 °C in a shaking incubator. For the test expression cultures, 50 μL of each starter culture was added to 50 mL LB with ampicillin in conical flasks. These were incubated at 37 °C until an OD600 of 0.6 was reached. The temperature was then reduced to 20 °C and protein expression was induced by adding 200mM IPTG and incubating overnight. Cells were harvested by centrifuging at 4000 rpm for 10 minutes. The supernatants were discarded and the pellets resuspended in 5 mL lysis buffer (50 mM Tris pH 8.5, 500 mM NaCl, 5% glycerol, 10 mM imidazole, 0.5 mM TCEP) with or without 0.5% CHAPS. Cells were lysed by sonicating for 1 minute with 10 second pulses. 5 μL lysate was set aside for the total protein fraction for the SDS-PAGE gel. The lysate was centrifuged at 4000 rpm for 15 minutes then 15 μL supernatant was set aside as the soluble protein fraction for the SDS-PAGE gel. The recombinant protein was separated by washing it through Amintra Ni-NTA resin in lysis buffer so the His tag would bind to the nickel. The flow-through was discarded then the recombinant protein was eluted from the nickel beads using elution buffer (lysis buffer with 250 mM imidazole) with or without CHAPS and collected in Eppendorf tubes. 15 μL of each elution was set aside and loaded onto on an SDS-PAGE gel with the total and soluble protein fractions for each test expression. 5 μL loading dye was added to each sample and after boiling at 98 °C for 1 minute, the samples were loaded onto a precast 4-12% gel alongside PageRuler protein ladder and a potential difference of 190 V was applied until the loading dye reached the bottom of the gel.

Large scale KRAS production: pLysS cells without CHAPS was selected for the large scale expression and purification of the KRAS protein. For the starter culture, 50 mL LB with ampicillin in a conical flask was inoculated with cells scraped from pLysS KRAS1-169 glycerol stocks made

from the test expression starter cultures. The flask was incubated overnight at 37 °C in a shaking incubator. 5 mL of starter culture was added to each of four large flasks containing 500 mL LB with ampicillin. Uniformly ^{15}N labelled KRAS samples were grown in modified Spizizen's media with 1.0 g/L of ^{15}N ammonium chloride as the sole nitrogen source. Flasks were incubated at 37 °C until OD600 of 0.6 was reached then the temperature was reduced to 20 °C and protein expression was induced with 200 mM IPTG. Flasks were then incubated overnight in a shaking incubator. The 2 L culture was combined and poured into two large centrifuge pots and centrifuged at 4000 rpm for 15 minutes. The supernatant was discarded and the pellets combined and resuspended in 25 mL lysis buffer with 50 µL DNase and one EDTA-free protease inhibitor tablet. The cells were lysed by sonicating for 10 cycles of 20 second pulses followed by 20 seconds off. The lysate was transferred to ultracentrifuge pots and centrifuged at 18000 rpm for 45 minutes.

Ni-NTA His purification: For the large scale expression of KRAS protein, the protein was separated via its His tag using a 5 mL nickel column attached to an AKTA Prime purifier. 50 mL supernatant was placed in a sample loop and connected to the AKTA Prime. The column was equilibrated with lysis buffer until the conductivity plateaued, after which a method with an increasing elution buffer gradient was used to elute the His-tagged protein and fractions were collected at regular intervals. The fractions which corresponded to the UV peak were collected and samples were loaded onto on an SDS-PAGE gel to determine which contained KRAS1-169 protein. This was repeated with the remainder of the supernatant and the relevant fractions were pooled together in a 50 mL falcon tube. 50 µL 1 mg/mL TEV protease was added and incubated in the fridge overnight to cleave the His tag.

For subsequent expressions, the addition of an autosampler to an AKTA Pure protein purification system (GE Healthcare Life Sciences) enabled Ni-NTA His purification without a sample loop.

Gel filtration: The cleaved protein was concentrated down to the required volume for the gel filtration column by centrifuging at 4000 rpm in a 1000 MWCO concentrator (Millipore) until there was less than 5 mL left. The concentrated protein was injected onto a Superdex 75 (10/300) gel filtration column attached to the AKTA Pure protein purification system which had been equilibrated overnight with the gel filtration buffer (20 mM Tris pH 8.5, 250 mM NaCl, 5% glycerol, 0.5 mM TCEP). The fractions which corresponded to the UV peak were collected and 10 µL of each fraction was analysed on an SDS-PAGE gel to check that it contained the cleaved KRAS1-169 protein. The relevant fractions were pooled then the protein concentration was determined using a Nanodrop 2000 spectrophotometer. The concentrated protein was then flash frozen and stored at -80 °C.

NanoMIPs synthesis:

NanoMIPs were produced using a recombinant protein (described above) or a peptide template immobilised on glass beads. A solid-phase approach described by Canfarotta *et al.* (7) was adapted for MIP nanoparticle synthesis. The surface of glass beads (200 g) was activated by boiling in sodium hydroxide (4 M, 160 mL) for 15 minutes prior to washing with water (3 x 200 mL). The beads were subsequently placed in a solution of sulphuric acid (50%, 160 mL) for 30 minutes before again washing with water (3 x 200 mL) and buffer (PBS, 3 x 200 mL), ensuring the final pH was between 6 – 8. Further washing with acetone (3 x 200 mL) was performed before drying under vacuum and placing the beads in an oven (150 °C) for 30 minutes. Activated beads were incubated in a solution of toluene (80 mL, anhydrous) with (3-aminopropyl)triethoxysilane (1.6 mL) and 1,2-bis(triethoxysilyl)ethane (0.27 mL) overnight at 70 °C. Beads were subsequently washed with methanol (3 x 200 mL) and acetone (5 x 200 mL) to remove any residual silane, before drying under vacuum and further oven drying for 30 minutes at 150 °C.

KRAS4A Sequence

```
          10         20         30         40         50
MTEYKLVVVG AGGVGKSALT IQLIQNHFVD EYDPTIEDSY RKQVVIDGET
          60         70         80         90        100
CLLDILDTAG QEEYSAMRDQ YMRTGEGFLC VFAINNTKSF EDIHHYREQI
         110        120        130        140        150
KRVKDSEDVP MVLVGNKCDL PSRTVDTKQA QDLARSYGIP FIETSAKTRQ
         160        170        180
RVEDAFYTLV REIRQYRLKK ISKEEKTPGC VKIKKCIIM
```

Figure S1 Amino acid sequence of KRAS4A protein. Blue box indicates C-terminus end and red box indicates area of N-terminus that contains G12 mutation.

KRAS peptides immobilisation:

A synthetic peptide (CTPGCVKIKKCIIM) was synthesised by Genecust and acted as the template (See Figure S1 for position of peptide). The KRAS C-terminal peptide template had an additional cysteine at the N-terminus to enable it to be immobilised on the glass beads in the correct orientation via thiol coupling. The cross-linker, N-succinimidyl iodoacetate (SIA), was used to link the template to the glass beads via the iodoacetyl group, which reacts with the sulfhydryl group on the cysteine to form a stable thioether bond and the NHS ester reacts to form a stable amide bond with the $–NH_2$ group on the glass beads. 130 g activated glass beads were weighed out and covered with 50 mL acetonitrile. 10 mg of SIA were added and the beads were left in the dark for two hours. Beads were washed with acetonitrile 5 times then dried under vacuum for 5 minutes. Oxygen was removed by vacuuming the beads in a desiccator then pumping with nitrogen and repeating 3 times. 50 mL PBS with 5 mM EDTA at pH 8.2 was added to the beads whilst bubbling

with nitrogen. 5 mg of the template peptide was dissolved in PBS/EDTA and added to the beads. Beads were incubated overnight in the dark in a sealed Duran bottle, swirling occasionally.

NanoMIP production: Polymerisation mixture consisting of NIPAM (39 mg), BIS (8 mg), TBAm (33 mg dissolved in 1 mL EtOH), AAc (100 µL of a 22 µL/1 mL solution in water) and APMA (5.80 mg) was prepared in water (100 mL) and purged with nitrogen for 30 minutes. Following this, the polymerisation mixture was added to the template derivatised beads (60 g) and polymerisation initiated using a solution of APS (30 mg/500 µL water) and TEMED (30 µL). The polymerisation was allowed to proceed for 60 minutes, before quenching of the reaction by allowing oxygen into the system. The beads were subsequently washed with distilled water (9 x 30 mL) at room temperature to remove unreacted monomer and low affinity polymer before eluting high-affinity nanoparticles with hot HPLC water (100 mL, 60 ˚C).

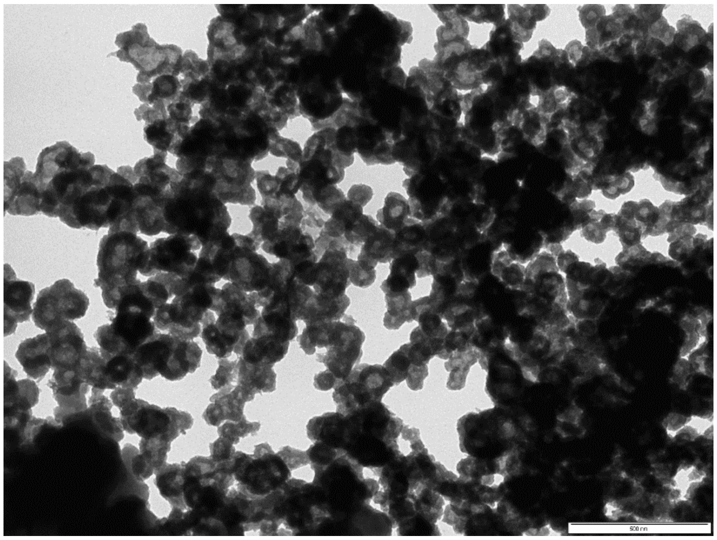

Figure S2. TEM image of KRAS-imprinted nanoMIPs. Scale bar is 500 nm.

TEM imaging: samples for TEM were prepared by placing 10 µL of the nanoMIP dispersion, previously sonicated for 5 min, onto a carbon coated copper grid. The sample was left to dry

overnight under a hood before imaging. Images were acquired using a JEOL JEM-2100 LaB6 TEM.

NanoMIP epitope discovery: firstly, the monomeric mixture was prepared using 19.5 mg of NIPAm and 3 mg of BIS in 50 mL PBS. 15 mg of TBAm were dissolved in 200 µL absolute ethanol and added to the monomeric mixture, followed by 50 µL acrylic acid and 3 mg N-(3-aminopropyl) methacrylamide hydrochloride. The mixture was sonicated and purged with nitrogen for 20 minutes. 2 mg of recombinant KRAS protein were added to 10 mL monomeric mixture and purged with nitrogen for 5 minutes. Polymerisation was initiated by adding 100 µL of an initiator solution prepared using 12 mg of potassium persulphate and 6 µL of N,N,N′,N′-tetramethylethylenediamine (TEMED) dissolved in 400 µL of PBS immediately before addition. The solution was left to polymerise for one hour at room temperature. The sample was then filtered through a 50 kDa MWCO centrifugal filter for 15 minutes at 4000 rpm. The sample was washed with 10 mL PBS to remove unreacted monomer and low affinity MIPs. 0.2 mg trypsin (trypsin from bovine pancreas, Sigma) in 5 mL PBS was added to the sample and incubated at room temperature for 96 hours. Unbound KRAS peptides and trypsin were removed by centrifuging for 5 minutes at 4000 rpm then washing twice with 10 mL PBS. The peptides bound to the MIPs were eluted using 1 mL hot water (heated to 95 °C). These potential epitopes were collected by centrifugation on 20 kDa MWCO filters. The hot wash was repeated and both elutions were combined, lyophilised and reconstituted in 20 µL 0.1% formic acid/3% acetonitrile and 20 µL 100 fmol/µL alcohol dehydrogenase (ADH). The samples were analysed in triplicate on a Waters Synapt G2 mass spectrometer coupled to a Waters Acquity nano-UPLC as described below.

Peptide MIPs characterisation

MIPs were characterised using a Zetasizer instrument (Malvern Instruments, UK) to determine the average diameter of the particles using dynamic light scattering (DLS). The Stokes-Einstein equation (Equation S1) is then used to calculate particle size. To mitigate potential issue with particle agglomeration, the solution was sonicated and passed through a 0.45 μm filter to separate the MIPs and remove large particles before measurement.

$$D = \frac{\kappa T}{6\pi\eta R}$$

Equation S1. The Stokes-Einstein equation where D is the translational diffusion coefficient (Brownian motion), k is Boltzmann's constant, T is the temperature of the fluid, R is the particle radius and η is the viscosity.[30]

Western blot

Antibodies used for western blots were anti-K-RAS AB180772 (lot GR3264774-1) from Abcam (Cambridge, UK) and anti-rabbit 7074s lot 26 from Cell Signalling Technology (London, UK).

Western blots were performed using an anti-KRAS antibody to measure KRAS in plasma samples. The aim was to compare the sensitivity of the MIP enrichment and mass spectrometry method to an established method. In the initial test blot, the four NSCLC samples with much greater peak areas for WT KRAS than the other samples were selected for analysis. These were CRB015, CRB016, CRB018 and CRB038. In addition, pancreatic cancer sample C3, which had the highest level of KRAS compared to the other pancreatic cancer samples, NSCLC sample CRB007 which had a known G12C mutation, and a healthy control plasma sample were analysed.

HCT-N and HT-29 cell lines were used as positive controls. Cells were lysed as follows. For the lysis buffer, one complete mini protease inhibitor tablet and one PhosSTOP phosphatase inhibitor tablet were dissolved in 10 mL RIPA buffer. The culture media was removed from the adherent cells which were then washed twice with PBS. Cells were incubated in Trypsin/EDTA solution until detached from the flask. Cells were resuspended in fresh media and centrifuged at 1300 rpm for 3 minutes in a centrifuge tube. The supernatant was removed then cells were resuspended in cold PBS and washed a subsequent two times. The PBS was removed and the cell pellet was resuspended in two pellet volumes of lysis buffer. Cells were incubated for 10 minutes on ice with intermittent vortexing to lyse the remaining cells.

A BCA assay was carried out to determine the concentration of protein in the plasma and cell lysate then 150 μg protein was loaded onto a gel for each sample. 20 mL 12% resolving gel was prepared using 6.8 mL water, 5 mL 4x Protogel resolving buffer, 8 mL 30% acrylamide, 0.2 mL 10% ammonium persulphate and 0.015 mL TEMED. The gel was mixed by inverting the tube then poured into the cast up to 2.5 cm below the top. Water was added to the top to ensure even setting of the gel. After 30 minutes, the water was removed. 10 mL 5% stacking gel was prepared with 5.7 mL water, 2.5 mL 4x Protogel stacking buffer, 1.7 mL 30% acrylamide, 0.1 mL 10% ammonium persulphate and 0.015 mL TEMED. The stacking gel was pipetted on top of the resolving gel. A 10 well comb was added and the gel was left to set for 60 minutes.

150 μg protein from each sample were mixed with an equivalent volume of 2x Laemmli buffer. Samples were heated at 100 °C for 5 minutes then quenched on ice and pulse spun. 1x running buffer was prepared by diluting 10x Tris Glycine SDS running buffer with water. Once set, the gel was placed in the gel running apparatus, the comb was removed and the gel was covered with running buffer. 5 μL molecular weight marker were loaded into the first well and the samples were

loaded into the remaining wells. The lid was connected and a constant voltage of 120 V was applied until the loading dye front reached 10 mm from the bottom of the gel.

1x transfer buffer was prepared by diluting 100 mL 10x transfer buffer in 200 mL methanol and 700 mL water. The gel was removed from the gel running apparatus and the gel plates were prised off the gel. The stacking gel was discarded and the remaining resolving gel was equilibrated in 1x transfer buffer for 10 minutes. A nitrocellulose sheet, two pieces of filter paper and two sponges were soaked in transfer buffer before being placed in the transfer cassette with the gel and the nitrocellulose sandwiched between filter paper and sponges. The gel was placed on the black side of the cassette and the cassette was inserted into transfer tank with black side connected to the black electrode. An ice block was added then the tank was filled with 1x transfer buffer. The lid was closed and 100 V current was applied for 60 minutes. The transfer buffer was removed and the nitrocellulose was carefully removed from the cassette and placed protein side up in a PerfectWestern container. To check the transfer was successful, a small amount of Ponceau S stain was added then removed and the nitrocellulose was washed with water so that the protein bands could be visualised.

For probing the western blot, washing buffer was prepared using 0.1% Tween20 in PBS (PBST). All washing, blocking and probing incubation steps were carried out using a rocking platform to ensure the blot didn't dry out. The nitrocellulose blot was washed in PBST for 5 minutes. Blocking solution was prepared using 5% Marvel non-fat milk powder in PBST and the blot was blocked for one hour at room temperature. The blot was then washed in PBST for 5 minutes. The anti-KRAS primary antibody was prepared as a 1:500 dilution in 10 mL 3% Marvel non-fat milk in PBST. The blot was incubated with the antibody for 90 minutes at room temperature. The antibody solution was then removed and the blot was washed with PBST for 10 minutes and twice for 5

minutes. The anti-rabbit secondary antibody was prepared as a 1:5000 dilution in 3% Marvel non-fat milk in PBST and incubated with the blot for 60 minutes at room temperature. The antibody solution was removed and the blot was washed once with PBST for 10 minutes and once for 5 minutes. This was followed by 5 minutes washing in water to remove the Tween20. Equal amounts of reagents one and two from the EZ-ECL detection kit were mixed and equilibrated for 5 minutes. The water was removed from the blot then 5 mL detection reagent was added and incubated for 2 minutes at room temperature. Excess ECL reagent was removed then the Syngene gel documentation system was used to image the blot.
